# The Role of ABCG2 in the Pathogenesis of Primary Hyperuricemia and Gout—An Update

**DOI:** 10.3390/ijms22136678

**Published:** 2021-06-22

**Authors:** Robert Eckenstaler, Ralf A. Benndorf

**Affiliations:** Institute of Pharmacy, Martin Luther University Halle-Wittenberg, 06120 Halle, Germany; robert.eckenstaler@pharmazie.uni-halle.de

**Keywords:** gout, early-onset gout, hyperuricemia, urate, uric acid, ABCG2, BCRP, ABC transporter, single nucleotide polymorphism, SNP

## Abstract

Urate homeostasis in humans is a complex and highly heritable process that involves i.e., metabolic urate biosynthesis, renal urate reabsorption, as well as renal and extrarenal urate excretion. Importantly, disturbances in urate excretion are a common cause of hyperuricemia and gout. The majority of urate is eliminated by glomerular filtration in the kidney followed by an, as yet, not fully elucidated interplay of multiple transporters involved in the reabsorption or excretion of urate in the succeeding segments of the nephron. In this context, genome-wide association studies and subsequent functional analyses have identified the ATP-binding cassette (ABC) transporter ABCG2 as an important urate transporter and have highlighted the role of single nucleotide polymorphisms (SNPs) in the pathogenesis of reduced cellular urate efflux, hyperuricemia, and early-onset gout. Recent publications also suggest that ABCG2 is particularly involved in intestinal urate elimination and thus may represent an interesting new target for pharmacotherapeutic intervention in hyperuricemia and gout. In this review, we specifically address the involvement of ABCG2 in renal and extrarenal urate elimination. In addition, we will shed light on newly identified polymorphisms in ABCG2 associated with early-onset gout.

## 1. Introduction

Gout is the clinical manifestation of hyperuricemia which is triggered by urate precipitation (deposition of monosodium urate crystals) in the synovial fluid of joints and other tissues [1,2]. The disease is primarily associated with severe arthropathy, which manifests mainly in the metatarsophalangeal joints (podagra), but also in other joints of the foot, ankles, knee, wrist, fingers, and elbows [3]. In the pathogenesis of the disease, urate deposits promote inflammatory responses in the synovial membrane (synovitis) and thus arthritis characterized by sudden, severe attacks of pain, swelling, redness, and tenderness in the affected joints. Depending on the course of the disease, the symptoms of gout can occur both as acute episodic flares (gout attacks) and persist chronically and, if left untreated, can lead to irreversible deformations and impaired mobility of the affected joints [3]. In addition, gout nephropathy, a form of chronic tubulointerstitial nephritis, induced by the deposition of urate precipitates in the distal collecting ducts and the medullary interstitium may cause progressive chronic kidney disease [4]. Furthermore, gout and hyperuricemia have been associated with a subset of comorbidities including metabolic syndrome, diabetes, hypertension as well as cardiovascular and cerebrovascular disease [5,6,7,8,9,10,11,12,13]. In most patients, the onset of gout occurs after the age of 60, with the incidence being about three times higher in men than in women [14]. However, a significant proportion of patients develop primary hyperuricemia and gout symptoms before the age of 40, which is defined as the pathotype of early-onset gout [15,16]. In addition to environmental factors, genetic predispositions leading to chronic, yet asymptomatic hyperuricemia in childhood and adolescence are considered to be the main causes for the early onset of the disease. Although not every patient with hyperuricemia necessarily develops gout [17], it is considered to be the major risk factor for the development and progression of the disease. In this review, we will specifically address the pathogenesis and genetic background of early-onset gout and highlight the role of intestinal uric acid transport in this context.

## 2. Gout and Hyperuricemia

Hyperuricemia represents a prolonged pathophysiological increased serum urate concentration, often defined as >6.0 mg/dL (>360 µmol/L) for females and >7.0 mg/dL (>420 µmol/L) for males [1,18], which is either caused by an increased hepatic biosynthesis or a reduced renal or intestinal excretion of urate [19]. Under physiological conditions, urate is derived from the enzymatic degradation of purine nucleobases/nucleotides, which are involved in a multitude of biochemical processes, such as energy metabolism and the formation of RNA and DNA [20]. In humans, urate is the terminal metabolite of purine catabolism derived from purines that do not enter the salvage pathway for the resynthesis of ATP or GTP [19]. Therefore, secondary hyperuricemia can be induced by an excessive intake of purine-rich food (e.g., red meat, offal, seafood) [21], cellular degradation processes, and high cell turnover in the context of leukemia/lymphoma [22] or anticancer treatment with chemo- or radiation therapy [23], which all increase the availability of free purines. In addition to a diet high in purines, other lifestyle-related behaviors such as excessive intake of fructose [24,25] and alcohol abuse [26,27] can also trigger hyperuricemia, which explains the high prevalence in industrialized countries and the increasing prevalence in developing countries [28]. Aside from the aforementioned environmental factors, also genetic defects in enzymes responsible for the biotransformation of purine bases can favor primary hyperuricemia, as is the case in Lesch–Nyhan or Kelley–Seegmiller syndromes [29]. In line with this notion, the heritability of hyperuricemia is substantial, suggesting important genetic contributions to urate homeostasis [30]. In pharmacotherapy, uricostatic drugs like the xanthinoxidase inhibitor allopurinol can be used to normalize hyperuricemia by preventing the last step in urate biosynthesis. Under this treatment, intermediates of the purine metabolism such as inosine, hypoxanthine, and xanthine accumulate, yet exhibit a better water solubility and a lower tendency to form crystals than urate. Unlike secondary hyperuricemias that are triggered by increased urate biosynthesis, the vast number (>90%) of primary hyperuricemia cases result from a decreased ability of the kidney or intestine to excrete urate [31]. The majority of urate (roughly 70%) is eliminated by the kidney, where it is freely filtered by the glomerulus [32]. Urate homeostasis is primarily influenced by renal proximal tubule cells, which express several transporters that either reabsorb urate (e.g., URAT1 at the apical and GLUT9 at the basolateral membrane) [33,34,35,36] or are involved in urate excretion (e.g., NPT1/4 at the apical and OAT1/3 at the basolateral membrane) [20,35,37,38]. Indeed, uricosuric drugs such as the URAT1 inhibitors benzbromarone as well as probenecid and lesinurad are used in pharmacotherapy to treat hyperuricemia by inhibiting renal reabsorption of urate [39]. In addition to transporters of the salute carrier (SLC) and the organic anion transporter (OAT) protein families, ABC transporters such as ABCG2 and ABCC4 are also involved in urate excretion [32,37]. As the previously mentioned other transporters, ABCG2 was shown to be located in the apical brush border membrane of renal proximal tubule cell [40]. In the intestine, the major site for the remaining 30% of urate excretion, the mechanisms of urate excretion are less well defined [38]. Urate transporters GLUT9 [41] and in particular ABCG2 [42] are highly expressed in intestinal epithelial cells and may thus represent interesting new pharmacological targets for the treatment of hyperuricemia [43,44,45,46,47]. Nonetheless, with regard to the sites of urate excretion (kidney & intestine) and the complex interplay of transporter-mediated excretion and reabsorption of urate in the kidney, the mechanisms of urate homeostasis are still not fully understood. However, single nucleotide polymorphisms (SNPs) in different genes involved in urate transport have been associated with hyperuricemia [48], thereby emphasizing the multicausal complexity of gout pathology [49]. In this article, we aim to focus on ABCG2, which has been identified as an important urate transporter in the intestine and the kidney [40,50,51,52], and discuss its role in renal and extra-renal urate excretion as well as in primary hyperuricemia and early-onset gout.

## 3. ABCG2 and Its Function in Renal Urate Elimination

**Figure 1:**
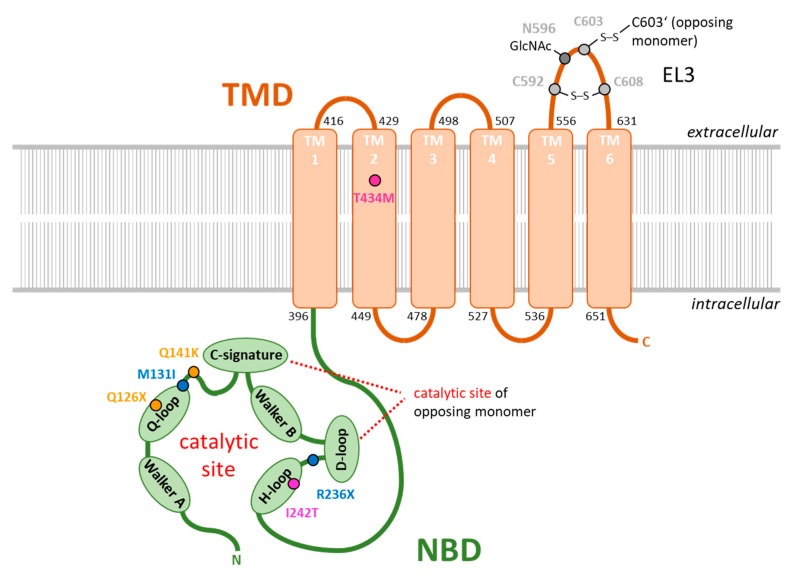
Polymorphisms in ABCG2 protein sequence associated with pediatric-onset hyperuricemia and early-onset gout. Schematic overview of the ABCG2 domain structure consisting of a nucleotide-binding domain (light green, NBD) and a transmembrane domain (light brown, TMD) modified from [69]. Single membrane-spanning α-helices (TM1–6) were structured according to the information of published protein sequences (NCBI accession number: NP_001335914.1). The catalytic site for ATP hydrolysis is formed by the sequence motifs Walker A, Q-loop, Walker B, and H-loop of one monomer, and the c-signature and D-loop from the other monomer. Cysteine bridge forming residues and N-acetylation sites within extracellular loop 3 (EL3) are marked in grey. SNPs involved in pediatric-onset hyperuricemia and early-onset of gout published in recent seminal publications are highlighted in different colors (yellow, dark blue, and purple).

ABCG2 (also known as BCRP) is a multi-drug efflux pump that has been described to contribute to transport processes in many different tissues and cell types. It belongs to the ABC (ATP-binding cassette) transporter superfamily [53] and has the ability to transport a variety of substrates across the membrane [54,55]. ABCG2 is highly expressed in the placental syncytiotrophoblasts [56], but can also be found at the entry and exit point of the human body including endothelial cells of the cerebral blood-brain barrier [57] and canalicular membrane of the liver [58] as well as polar epithelial cells of the intestine [42] and the kidney [50,59]. Based on its function and localization, ABCG2 is thought to act as a “gatekeeper”, preventing endo- or exotoxins and xenobiotics from crossing biological barriers and entering sensitive tissues [60]. Although these functions of ABCG2 are thought to serve to maintain the healthy state of the organism, they also appear to be responsible for ABCG2-related interference with pharmacotherapeutic interventions to treat certain diseases. In this regard, overexpression of ABCG2 has been associated with multidrug resistance to chemotherapy [61,62], which is associated with poor prognosis in the treatment of certain cancers [63,64]. With regard to its protein structure, ABCG2 consists of an ATP-hydrolyzing nucleotide-binding domain, which is located in the cytoplasm and provides energy for the transport process, and a transmembrane domain, which is responsible for the binding of substrates and their transport across the membrane (Figure 1). Moreover, ABCG2 is a so-called “half-transporter” that needs to homodimerize to form a functional transporter [60]. Recently, several high-resolution 3D structures of the ABCG2 protein bound to different substrates and inhibitors have been solved [65,66,67,68], which help to understand the molecular mechanisms of substrate selection, substrate binding, and substrate transport of ABCG2 [69,70]. In addition to its role as an efflux pump with broad specificity, ABCG2 has been proposed to be involved in renal and intestinal urate excretion [40,50,51,52,71]. The function of ABCG2 as a urate transporter was inferred from genome-wide association analyses and subsequent functional studies, which specifically demonstrated a strong association of a missense SNP in the ABCG2 gene (rs2231142; Q141K) with hyperuricemia [72,73,74], an SNP that could be causally related to at least 10% of all gout cases [50]. The Q141K polymorphism has been associated with a reduced ABCG2 surface expression and decreases cellular urate efflux to approximately half of wild-type ABCG2 levels [50,52,75,76,77]. In structural predictions derived from homology models [78] as well as structural cryo-EM data [65], Q141 was shown to be located in the nucleotide-binding domain of the transporter and to form a hydrogen bond to N158 of an α-helix within the nucleotide-binding domain adjacent to transmembrane helix 1 of ABCG2. This connection might be responsible to convey conformational changes induced by ATP binding or ATP hydrolysis from the nucleotide-binding domain to the substrate transporting transmembrane domain, thereby potentially explaining the partial loss of function of the Q141K-mutated transporter. However, also misfolding, reduced protein stability, and reduced membrane expression due to increased proteasomal degradation of Q141K-mutated ABCG2 [75,79] are also discussed as causes of the urate excretion deficit. Recent findings suggest that Q141K- and M71V-related dysfunction is due to aberrant trafficking of ABCG2 to the plasma membrane due to quality control mechanisms in the endoplasmic reticulum rather than reduced ABCG2 transport activity [80]. Moreover, the c.C421 > A mutation that leads to the Q141K polymorphism promotes microRNA-mediated suppression of ABCG2 translation, so that cell type-specific processing of the ABCG2 3`UTR along with cell type-specific microRNA expression profiles may have a profound impact on functional ABCG2 bioavailability in individuals carrying the Q141K polymorphism [76]. In the kidney of humans and mice, ABCG2 was shown to be expressed in the apical membrane of the brush border of proximal tubule epithelial cells [40,50], although in the analyses of The Human Protein Atlas consortium ABCG2 could not be detected at the protein level in human kidney biopsies [81]. Nonetheless, also findings from other groups indicate relevant renal ABCG2 expression [59]. The renal expression pattern of ABCG2 partially resembles the expression pattern of urate reabsorbing transporter URAT1, thereby indicating a functional interplay of both transporters in renal urate handling [40]. However, in measurements of renal urate excretion after a purine challenge (oral administration of inosine, which is rapidly metabolized to urate), human subjects carrying the ABCG2 transport function impairing Q141K polymorphism showed no significant differences in urate excretion and a fraction of filtered urate load (FEUA defined as the ratio between the renal clearance of uric acid to the renal clearance of creatinine), although their serum urate levels were significantly elevated [40]. In the same study, renal urate excretion was also investigated in an orthologous Q140K knock-in mouse model. Here, only the male animals displayed elevated serum urate levels and had, in contrast to humans, at least a significantly reduced fraction of filtered urate load but again no change in urinary urate excretion [40]. Interestingly, these sex-related phenotypes were consistent with the increased prevalence of gout in human males [28]. However, the results of both human and mouse experiments suggest that the hyperuricemia induced by the ABCG2 Q141K polymorphism is not caused by a significant effect on renal urate excretion, but is likely to be triggered by different mechanism [40]. These findings, which are in line with the ABCG2 expression data from The Human Protein Atlas consortium [81], also raise the fundamental question of whether ABCG2 is in fact of any significance for renal urate excretion. In this regard, conflicting results regarding the involvement of ABCG2 in renal urate elimination have been obtained in experiments with ABCG2 knockout mice [51,71]. In both studies, serum urate concentrations of ABCG2 knockout animals were elevated compared to their wildtype littermates. However, while one study did not observe significant differences in renal urate elimination [71], the other study found a significant reduction of about 30% [51]. Nevertheless, these two animal studies, as well as the translational study by Hoque and colleagues, indicate that ABCG2 primarily affects extrarenal regulation of urate homeostasis, which is further discussed below. It should be mentioned that in the kidney, ABCG2 is only one of many renal transporters that are able to excrete urate [37] so that ABCG2 loss of function may be compensated by other transporters. In conclusion, the previously assumed relevance of ABCG2 for renal urate elimination has been questioned by recent studies and therefore further future studies are needed to definitively elucidate this issue.

## 4. Relevance of ABCG2 in Extrarenal Urate Elimination

As earlier studies indicated that ABCG2 is also expressed in the liver [58] and intestine [42] and that ABCG2 may function as a urate transporter [50,52], it was reasonable to speculate that ABCG2 plays a role in extrarenal urate excretion possibly via the bile or intestine [82]. However, accurate non-invasive measurements of intestinal/biliary urate secretion are not possible in humans because the secreted urate is largely metabolized by the bacterial flora in the intestine. Therefore, the role of ABCG2 in extrarenal urate secretion was revealed in animal experiments using the in situ intestinal “closed-loop” perfusion method [51,71]. Since most vertebrates, including rodents used for animal studies, express the enzyme uricase which converts uric acid to allantoin and which has been lost during human evolution [83], a direct relation of results from animal experiments studying urate metabolism and urate transport to the human organism is inadequate. To account for that, rodents used in both studies were treated with the uricase inhibitor oxonate, a pharmacological intervention that increased the serum urate levels in mice and rats to the magnitude of serum urate levels in humans [51,71]. By administration of radioactive labeled uric acid, Hosomi and colleagues demonstrated that, in addition to the substantial fraction of renal urate elimination [51], there is direct urate excretion via the intestine (mainly in the ileum) and only minor urate excretion via the bile [51,71]. These findings, therefore, suggest that the intestine is the main site of extrarenal urate excretion. In the intestine of mice, ABCG2 expression is mainly located at the villi brush border of epithelial cells of the ileum and the jejunum [40]. Interestingly, in this study, the observed overall expression levels of the protein in the intestine are much higher than in the kidney. In rats, ABCG2 expression in the gut was found to further increase in response to increased blood urate concentrations after oxonate treatment [84]. To study the contribution of ABCG2 in intestinal urate excretion (and renal urate excretion which was already discussed above), oxonate-treated ABCG2 knockout mice were used [51,71] and showed a reduction in intestinal urate elimination by roughly 40–50%. These findings also suggest that other yet unknown transporters besides ABCG2 are involved in intestinal urate secretion [51,71]. A similar severe loss in intestinal urate elimination was also observed in the aforementioned study by Hogue and colleagues in an orthologous Q140K knock-in mouse model but in absence of the uricase inhibitor oxonate [40]. Consistent with these data, Q140K knock-in in mice resulted in a marked reduction in intestinal ABCG2 expression. In contrast, only subtle changes in urate elimination and ABCG2 expression were observed in the kidney in the same knock-in mouse model. These results are further supported by the observation of other authors, which indicate that the ABCG2 Q141K polymorphism and fractional renal clearance both contribute significantly but independently to the risk of hyperuricemia in humans [73]. In addition, impaired intestinal urate excretion induced by the orthologous murine Q140K mutation or complete ABCG2 knockout may explain hyperuricemia despite unaltered renal urate excretion in the respective mouse models [71,85,86] as well as in human individuals carrying the Q141K polymorphism [40]. However, due to the rise in serum urate levels (sUA) caused by the lack of intestinal urate secretion, an indirect increase in the fraction of filtered urate load (FEUA) could be expected in patients with ABCG2 dysfunction although this was not observed in the previously mentioned study [40]. In addition, ABCG2-mediated intestinal urate elimination appears to play an important role in compensating for the loss of renal urate elimination in chronic kidney disease [35]. Taken together, recent publications indicate that the major site of action of the ABCG2 transporter is regulating urate homeostasis in the intestine.

## 5. ABCG2 Polymorphisms in Pediatric-Onset Hyperuricemia and Early-Onset Gout

Hyperuricemia and gout pathology has often been shown to be related to genetic predisposition [30] and to be affected by SNPs in many of the genes encoding urate transporters [87,88]. Among these, especially SNPs of ABCG2 have been highly associated with pediatric-onset hyperuricemia and early-onset gout [89,90,91,92,93]. These polymorphisms are summarized and organized in Table 1 according to the standard nomenclature rules for molecular diagnostics [94]. Furthermore, the localization of these polymorphisms on the protein sequence of ABCG2 is shown in Figure 1. It should be noted that there is a subset of other function-impairing SNPs in ABCG2 [95,96], but most of them have not yet been associated with pediatric hyperuricemia or early-onset gout. One of the best-studied variations of the ABCG2 amino acid sequence is the previously discussed Q141K polymorphism, which also gives rise to other important clinical phenotypes, such as in the pharmacokinetics and tissue distribution of drugs transported by ABCG2 [97]. The polymorphism is highly associated with early-onset hyperuricemia, gout, and hyperuricemia-associated comorbidities, which cause a high mortality rate in hemodialysis patients [98]. Although the F489L polymorphism has not been as well studied in the context of disease, it shows a similar inhibitory effect on the ABCG2 transport function as the Q141K mutation. As with the Q141K mutation, ABCG2 carriers with the F489L mutation show reduced expression and reduced ABCG2 transport capacity [75]. Inhibition of proteasomal degradation could partially restore the transport function of both ABCG2 variants. In contrast to the Q141K polymorphism, which causes amino acid sequence alterations in the nucleotide-binding domain, the F489L polymorphism is localized in the transmembrane domain. This shows that the impairment of ABCG2 function can be caused by changes in amino acid structure in different domains of the transporter. Polymorphisms in the transmembrane domain have often been associated with decreased surface expression of the ABCG2 transporter and impaired substrate transport abilities [99]. However, there is not much literature to support their clinical impact in both late and early-onset hyperuricemia and gout. The clinical importance of a certain polymorphism on the development of hyperuricemia and gout usually is related to its minor allele frequency in humans and its functional impact on the protein of interest. Due to genetic drift caused by spatial separation of populations, certain polymorphisms have accumulated in different ethnicities. For example, the frequency of V12M polymorphism is high in Mexican Indians but low in Caucasian and Middle Eastern populations [97]. In contrast, the Q141K and Q126X polymorphisms are enriched in Japanese populations, whereas in Caucasians, Q141K is not as common and Q126X is virtually absent [97]. Our understanding of the genetic variations in the ABCG2 sequence associated with hyperuricemia and gout is still incomplete, as evidenced by the recent discovery of less common polymorphisms previously unrecognized or not studied in the context of hyperuricemia and gout [89,90,95,100]. Two of these newly identified rare polymorphisms have been recently described in a case report of a 12-year-old Czech girl of Roma ethnicity with chronic asymptomatic pediatric-onset of hyperuricemia [89]. In this regard, several rare diseases have been found to occur primarily or exclusively in individuals of Roma ethnicity, and many of the mutations underlying these diseases have been recently discovered, such as for Charcot-Marie tooth disease types 4D and 4G [101,102], the congenital cataract facial dysmorphism neuropathy [103], the Gitelman syndrome [104], and the Galactokinase deficiency [105]. In the afore-mentioned case of the 12-year old girl, DNA sequencing analysis of the ABCG2 gene revealed the presence of heterozygously expressed missense (c.393G > T, p.M131I) and nonsense (c.706C > T, p.R236X) mutations (Figure 1, blue residues) causing the pediatric-onset of hyperuricemia observed in the girl`s ancestry and the early-onset of gout especially in male individuals of the maternal line of inheritance. In the study, the functional consequences of the mutations were investigated in comparative in vitro experiments. Due to the in-frame stop codon induced by the R236X mutation, the ABCG2 protein sequence was truncated to about 1/3 of the full-length protein, with the mutant protein lacking a functional transmembrane domain. Therefore, no plasma membrane localization and no urate transport activity of the mutant protein could be observed. In contrast, the M131I mutation was translated to a full-length protein with no impairments in N-glycosylation at residue N596 and normal membrane localization. However, the urate transport capabilities of the M131I mutant were reduced to <15% of wildtype levels [89]. M131 itself was found to be a highly conserved residue that is localized close to the Q-loop within the nucleotide-binding domain of ABCG2 (Figure 1). The conserved glutamine Q126 in the center of the Q-loop is responsible for the coordination of the magnesium ion associated with ATP in the catalytic center of the protein [68]. M131I may thus alter the spatial orientation of the Q-loop or sterically hinder the coordination of Mg-ATP, thereby drastically reducing ABCG2′s ATP hydrolysis capabilities necessary for providing the energy for substrate transport across the membrane. Another newly identified polymorphism associated with pediatric hyperuricemia and early-onset gout is I242T, which was found in the lineage of another young European girl and was analyzed in a similar way [93]. Like the aforementioned M131I mutant, the I242T mutant ABCG2 variant showed no impairment in glycosylation and membrane localization, although its urate transport abilities were drastically reduced. This effect could be coincidentally related to the close localization of the mutants at the conserved H243 within the H-loop or also called histidine switch of the catalytic center of ABCG2 (Figure 1). The H-loop is responsible for coordinating the γ-phosphate of ATP, which is responsible for ATP hydrolysis. For further research, I242T and M131I may represent interesting new candidates to study the consequences of ABCG2 loss-of-function without disrupting ABCG2 membrane localization and protein-protein interactions. These representative case reports also show that depending on the severity of the disruption of the urate transportability of ABCG2, homo- or heterozygosity of the dysfunctional polymorphisms and further genetic predispositions in other genes involved in urate homeostasis [106], hyperuricemia can already occur in childhood (pediatric-onset), which increases the risk for the development of early-onset gout. This allows the risk allele of a particular polymorphism to be identified and considered for clinical diagnosis. Interestingly, compared to patients with late-onset gout, patients with early-onset gout also show clinical symptoms that indicate a more severe disease pattern. This includes a prolonged disease duration, a different localization of the first occurring arthritis (with a lower incidence of typical metatarsophalangeal manifestations and a higher incidence of ankle- or mid-foot involvement in early-onset gout), a higher flare frequency (gout attacks), and an increased overall number of involved joints [15,16]. In terms of gout-associated comorbidities, late-onset gout patients are more likely to suffer from chronic kidney disease, metabolic syndrome, and cardiovascular disease, a phenomenon probably related to the age difference between the two patient groups [16]. However, these comorbidities occur at a younger age in patients with early-onset gout. In contrast, a recent study showed that patients diagnosed with gout at age 40 or younger may be at increased risk for cardiovascular disease and recurrent gout compared to those diagnosed later in life [107]. In this study, of 427 adult patients diagnosed with gout at a New England multispecialty group practice, 327 who were aged 40 years or younger at diagnosis were more likely to have cardiovascular risk factors. For example, these younger patients had a significantly higher body mass index than gout patients over 40 years of age, and a substantial proportion of the younger patients also suffered from hypertension or hyperlipidemia. Moreover, early-onset gout patients were less likely to achieve a serum uric acid level below 6.0 mg/dL after therapeutic intervention as compared to late-onset gout patients. Therefore, clinical screening for hyperuricemia in genetically predisposed families and prompt urate-lowering therapy in pediatric, adolescent, or young adult patients with still asymptomatic chronic hyperuricemia could help delay the onset of gout and the development of hyperuricemia-related comorbidities [108,109,110]. With regard to the treatment of cardiovascular comorbidities in hyperuricemia patients, it should be noted that blood pressure-lowering drugs such as the AT1 receptor blocker telmisartan have been shown to inhibit the transport activity of ABCG2 [75] thereby potentially exacerbating hyperuricemia in patients with a corresponding genetic predisposition. In view of the emerging role of ABCG2 and its importance for intestinal excretion of uric acid, it may in principle represent a novel pharmacotherapeutic target to lower uric acid levels [43,44,45,46,47]. As speculation, this may be accomplished by modifying ABCG2 expression and function in intestinal epithelial cells. For example, in patients expressing mutant forms of ABCG2, this opens up the possibility of developing small molecule drugs with high pre-systemic elimination to target the function, cellular handling, or expression of ABCG2 predominantly in intestinal epithelial cells, thereby locally normalizing the impaired intestinal uric acid excretion in these individuals without interfering with the function of ABCG2 in other tissues (e.g., extrusion of xenobiotics). This area of research, therefore, shows great potential for the development of targeted pharmacotherapies for specific populations of genetically predisposed individuals with early-onset gout and thus warrants innovative research in the near future.

## 6. Conclusions

Gout is a major health care burden in developed countries, where it affects about 1% to 2% of the adult population and is the most common cause of inflammatory arthritis in men. In addition to obesity and hyperuricemia, lifestyle changes that have developed in industrialized countries in recent decades, such as a diet rich in red meat and fructose, physical inactivity, and increased alcohol consumption, may play a role in the shift toward a younger age of manifestation of gout in the population and require early intervention. As there is evidence that early onset of hyperuricemia and gout is associated not only with a severe clinical course of gouty arthritis, but also with other comorbidities, such as hypertension, metabolic syndrome, and cardiovascular complications, early detection of hyperuricemia in younger patients with genetic predisposition and early uric acid-lowering therapy should be considered to reduce morbidity and mortality in these patients. 

## Figures and Tables

**Table 1:** Polymorphisms in ABCG2 protein sequence associated with pediatric-onset hyperuricemia and early-onset gout.

rs ID	Coding Sequence	Protein Sequence	Citation
rs72552713	c.376C > T	p.Q126X	[92]
rs759726272	c.393G > T	p.M131I	[89]
rs2231142	c.C421 > A	p.Q141K	[91,92]
rs140207606	c.706C > T	p.R236X	[89]
not annotated	c.725T > C	p.I242T	[93]
rs769734146	c.1301C > T	p.T434M	[90,100]

## Data Availability

Not applicable.
